# Quantification of wild-type and radiation attenuated *Plasmodium falciparum* sporozoite motility in human skin

**DOI:** 10.1038/s41598-019-49895-3

**Published:** 2019-09-17

**Authors:** Béatrice M. F. Winkel, Clarize M. de Korne, Matthias N. van Oosterom, Diego Staphorst, Mark Meijhuis, Els Baalbergen, Munisha S. Ganesh, Koen J. Dechering, Martijn W. Vos, Séverine C. Chevalley-Maurel, Blandine Franke-Fayard, Fijs W. B. van Leeuwen, Meta Roestenberg

**Affiliations:** 1 Department of Parasitology, Leiden University Medical Center, Albinusdreef 2, 2333 ZA, Leiden, The Netherlands; 2 Interventional Molecular Imaging laboratory, Department of Radiology, Leiden University Medical Center, Albinusdreef 2, 2333 ZA, Leiden, The Netherlands; 3 TropIQ Health Sciences, Transistorweg 5, 6534 Nijmegen, The Netherlands; 4 Department of Infectious Diseases, Leiden University Medical Center, Albinusdreef 2, 2333 ZA, Leiden, The Netherlands

**Keywords:** Parasitology, Infectious diseases, Preclinical research

## Abstract

Given the number of global malaria cases and deaths, the need for a vaccine against *Plasmodium falciparum* (*Pf*) remains pressing. Administration of live, radiation-attenuated *Pf* sporozoites can fully protect malaria-naïve individuals. Despite the fact that motility of these attenuated parasites is key to their infectivity and ultimately protective efficacy, sporozoite motility in human tissue (e.g. skin) remains wholly uncharacterized to date. We show that the ability to quantitatively address the complexity of sporozoite motility in human tissue provides an additional tool in the development of attenuated sporozoite vaccines. We imaged *Pf* movement in the skin of its natural host and compared wild-type and radiation-attenuated GFP-expressing *Pf* sporozoites. Using custom image analysis software and human skin explants we were able to quantitatively study their key motility features. This head-to-head comparison revealed that radiation attenuation impaired the capacity of sporozoites to vary their movement angle, velocity and direction, promoting less refined movement patterns. Understanding and overcoming these changes in motility will contribute to the development of an efficacious attenuated parasite malaria vaccine.

## Introduction

Nearly half the human population lives in areas with an increased risk of malaria transmission, resulting in more than 200 million cases each year^1^, illustrating the urgent need for a highly effective malaria vaccine. Vaccines based on live attenuated *Plasmodium falciparum* (*Pf*) parasites obtained from the mosquito salivary gland, so-called sporozoites, are currently most promising. Clinical trials that used mosquitoes to transmit the *Pf* parasites into the human subjects yielded 100% protection in non-endemic settings^2–4^. When translating the seminal mosquito-bite studies into a needle-based cryopreserved vaccine formulation^3^, intradermal (ID) injection of isolated live attenuated sporozoites, were found to induce inferior protective immunity in humans^5^. Further research showed that an impractical intravenous (IV) route of attenuated parasite injection was more effective, because IV administration promotes transportation of parasites to the liver^3,4,6^, which is key to the induction of protection^7,8^. To date, the factors which affect motility of *Pf* from human skin to liver have not been studied.

Imaging studies using genetically modified fluorescent *Plasmodium berghei (Pb*) or *yoelii* *(Py*) sporozoites in mouse skin yielded insight into the migration patterns of sporozoites, with contrasting differences between *in vitro* and *in vivo* motility or the site of injection (tail or ear)^9–14^. Because the anatomical structure of murine skin differs from human skin, with respect to thickness, muscle layers, dermal papillae and hair follicle density^15,16^, a human skin model would provide a valuable contribution. Despite differences such as the lack of blood flow, *ex vivo* skin explant models have shown excellent viability of dermal cells over long periods of time^17,18^. Analysis of *Pf* migration in human tissue is an important first step to understanding *Pf* transmission and attenuated parasite vaccine delivery. In addition, a human skin model could also allow for future evaluation of subunit vaccines^14^.

Attenuated sporozoite vaccines have been produced using radiation attenuation (RA), gene modification or concomitant drug administration^19^. RA is the most commonly used method, whereby RA sporozoite vaccines are currently entering phase 3 clinical trials^20,21^. RA introduces double strand breaks in DNA^22^ and has been shown to impact the sporozoites gene expression and ultrastructure^23–25^. In both ways, sporozoite motility might be influenced. At present, preservation of motility following RA can only be validated using an *in vitro* gliding assay^26^, where the sporozoite is allowed to glide on a glass surface. However, this assay does not mimic the complexity of environmental interactions that are observed in tissue (e.g. physical confinement)^10,27^. Therefore, *ex vivo* imaging technologies that make use of human tissue are required.

The pioneering literature that presents image analysis of rodent sporozoite (*Pb)* movement in murine tissue, has focused on an often used measure of random diffusion of particles, the mean squared displacement (MSD)^9,10,28^. This measurement separates anomalous diffusion (with a non-linear relation to time), from the classic linear diffusion process. This has supported quantitative investigations towards motility changes over time and in relation to dermal structures^9,10^. As sporozoite movement through tissue suits a specific purpose, it makes sense for motility analyses to include quantitative parameters for directionality rather than limit the analysis to parameters that present random diffusion. Analysis of directional movement, referred to as tortuosity, is a common approach to study e.g. animal migration through the desert^29^ and analysis of disease severity in cognitively impaired patients^30^, but has also been applied during cell tracking studies^31^. The tortuosity of movement indicates whether it is directional or random. We reasoned that the concept of directional movement could complement the diffusion-based in skin sporozoite analysis and could provide a more detailed insight in sporozoite motility in complex environments such as human skin tissue.

**Figure 1:**
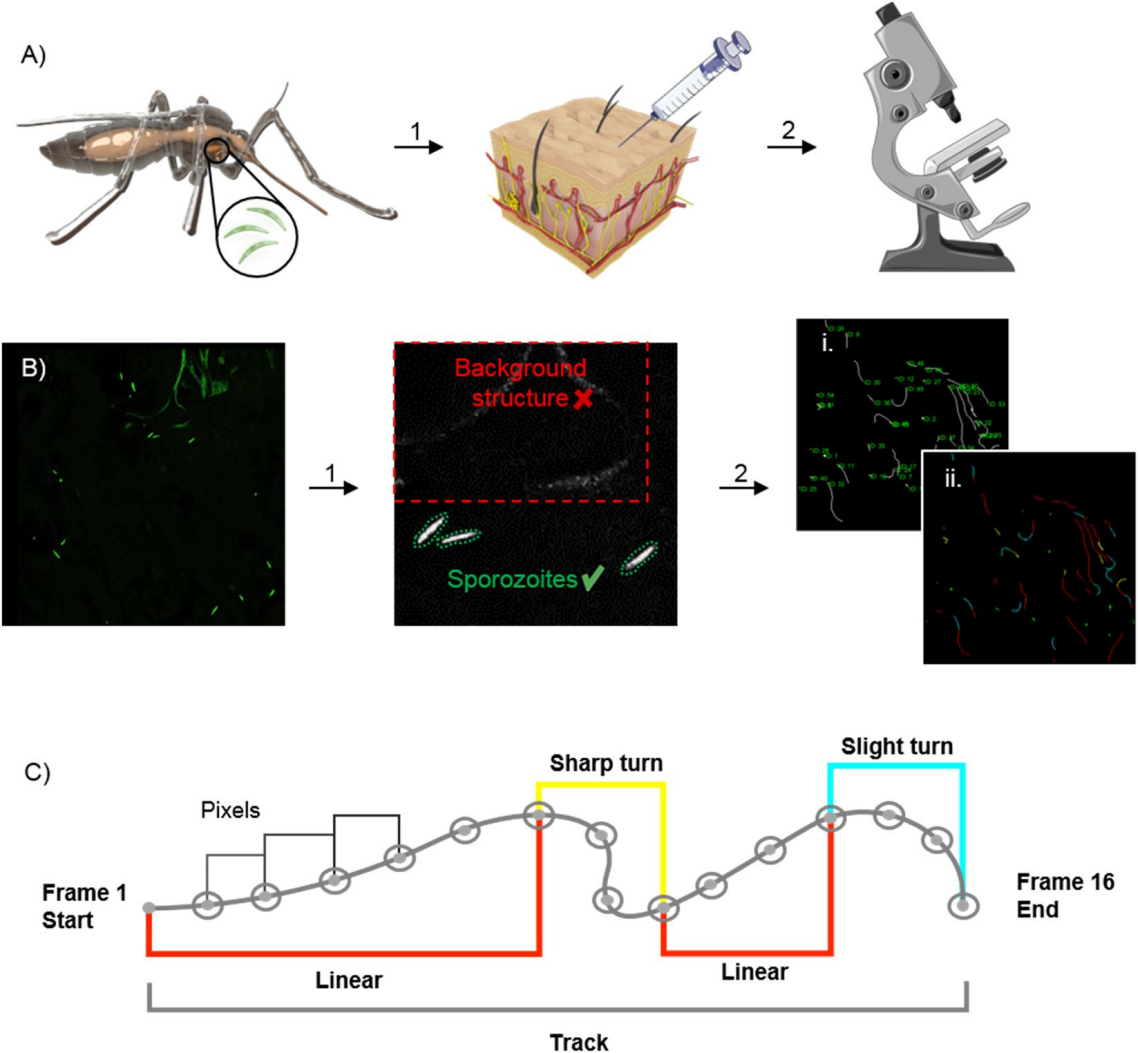
Schematic of experimental setup and SMOOT_human skin_ analysis. (**A**) Schematic of an *Anopheles* mosquito as the host of *Plasmodium* sporozoites within its salivary glands. Isolated sporozoites, *Pf*  ^WT^ or *Pf*  ^RA^, were injected into human skin (1). The skin samples containing sporozoites were filmed using a confocal microscope (2) (Images of needle and microscope were adapted from image copyright https://smart.servier.com, Creative Commons Attribution 3.0 Unported License, https://creativecommons.org/licenses/by/3.0/). (**B**) Raw confocal video images were uploaded into SMOOT_human skin_. Per video frame individual sporozoites were semi-automatically segmented (1). Segmented sporozoites in consecutive frames were stitched to generate tracks. Generated tracks have a unique sporozoite ID (i) in order to extract measured parameters (for example movement pattern (ii)) per sporozoite over time (2). (**C**) Sporozoite tracks are divided into segments based on the underlying movement pattern.

The aim of this study was to image and quantitatively assess motility of wild-type, GFP-expressing *Pf* sporozoites (*Pf*  ^WT^) in human skin (Fig. 1). For image analysis we developed a software tool for in skin analysis called SMOOT_human skin_ (Sporozoite Motility Orienting and Organizing Tool) that can output tortuosity and velocity related motility parameters of individual *Pf* sporozoites. In order to assess the effect of RA on sporozoite migration, we subsequently compared the motility of *Pf*  ^WT^ and RA sporozoite (*Pf*  ^RA^) populations in human skin explants(tail or ear).

## Results

### Generation of a semi-automated sporozoite migration analysis tool

Firstly, we generated confocal microscopy movies of *Pf*  ^WT^ and *Pf*  ^RA^ migrating through human skin explants by a trans sectional skin setup (fourteen 11 min movie sections of *Pf*  ^WT^ and eighteen 11 min movie sections of *Pf*  ^RA^, yielding a total of 352 and 350 analyzed sporozoites, respectively). Experiments were performed in two independent donor samples and while splitting the batch of sporozoites in a *Pf*  ^WT^ and *Pf*  ^RA^ group. Using our semi-automated software tool SMOOT_human skin_, we were able to track sporozoite movement (Fig. 1) by identifying sporozoites based on their shape and fluorescence intensity (Fig. 1B) and connecting their location over time (see supplementary figure S1 for an overview of the data per individual location and supplementary movie S1 and S2 for examples). Sporozoite locations per frame were stitched together in order to generate track segments, where multiple segments in the same 2D plane build up a full track (Fig. 1B, C). Depending on the straightness index (SI) of the individual segments, their movement patterns (sharp turn, slight turn and linear) were determined and color coded (Fig. 1C). Slight turns were defined as the turns which resulted from the natural curvature of the sporozoites (0,21–0,23 (1/µm)^32^. Sharper turns, requiring extra bending of the sporozoite, were defined as sharp turns^32^. Furthermore, the following movement parameters were calculated at track level: SI and angular dispersion (AD), at segment level: turn direction (clockwise or counter clockwise) and at frame level: MSD and velocity. Using the unique ID allocated to each individual sporozoite track, all computed parameters were extracted for the individual sporozoites and, where relevant, analyzed over time. In the experiments 81% of the sporozoites were characterized as motile, which surpasses the 66% reported earlier for *Pb* sporozoites in murine skin^9^.

### Mean squared displacement (MSD) data alone does not reflect individual sporozoite movement heterogeneity

**Figure 2:**
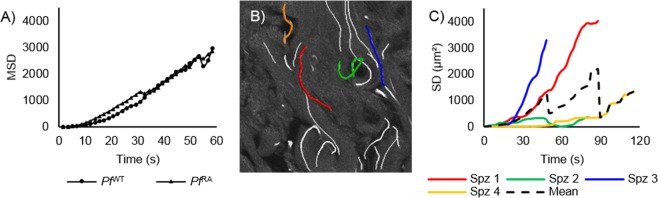
Mean squared displacement of sporozoites. (**A**) Mean squared displacement (MSD in µm^2^) of the sporozoites plotted against time, only linear tracks are taken into account. (**B**) Examples of individual sporozoite tracks in a single movie file. (**C**) The four differently colored individual tracks from B. are presented as squared displacement (SD in µm^2^) over time and presented relative to the dotted line, which presents the MSD of all four lines.

In analogy to previous protocols for *Pb* sporozoites^10^, we evaluated the MSD of linear *Pf*  ^WT^ tracks (37% of tracks). Comparing the MSD of *Pf*  ^WT^ with the *Pf*  ^RA^ population (containing 18.5% total linear tracks) yielded no significant difference (Fig. 2A). This analysis, however, excluded a large fraction of sporozoites that exhibited non-linear movement (slight and sharp turn; 81.5% for *Pf*  ^RA^ and 63% for *Pf*  ^WT^). We thus concluded that this analysis methodology was not suitable to fully grasp the complexity of *Pf* motility in human skin. In addition, we found that squared displacement plots of sporozoites revealed a high level of heterogeneity. A typical example of this heterogeneity is shown in Fig. 2B,C, where we plotted the squared displacement (SD; where displacement is the difference in sporozoite position between begin and endpoint of a track) of 4 very different individual sporozoite tracks from the same movie file. In order to do justice to the sample heterogeneity, we aimed to include other parameters of movement, such as the tortuosity, in the motility analysis.

### Tortuosity analysis reveals differences in sporozoite motility after RA

**Figure 3:**
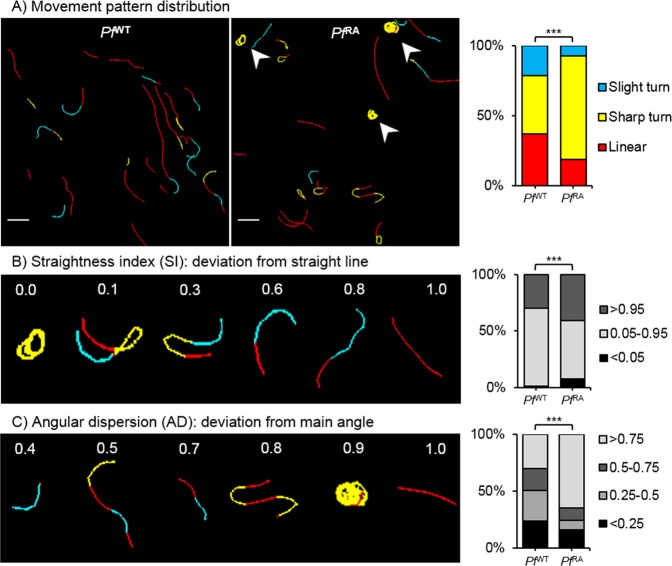
Tortuosity of sporozoite tracks. (**A**) Two examples of movement pattern maps of tracked sporozoites; *Pf*  ^WT^ in skin (left) and *Pf*  ^RA^ in skin (middle). Linear segments are depicted in red, slight turns in blue and sharp turns in yellow. Arrowheads indicate circular sporozoite tracks comparable to *in vitro* movement. The movement pattern distribution of the *Pf*  ^WT^ and *Pf*  ^RA^ is quantified for all sporozoite tracks based on frames (right). Scale bar: 20 µm. (**B**) To illustrate the concept of straightness index (SI) in relation to sporozoite tracks, 6 tracks are displayed out of the movement pattern maps shown in A. The SI distribution is quantified based on the SI values of total tracks (*Pf*  ^WT^ median: 0.89, IQR: 0.66–0.96; *Pf*  ^RA^ median: 0.90, IQR: 0.46–0.98). (**C**) To illustrate the concept of angular dispersion (AD) in relation to sporozoite tracks, 6 tracks are displayed out of the movement pattern maps shown in A. The AD distribution is quantified based on the AD values of total tracks (*Pf*  ^WT^ median: 0.47, IQR: 0.26–0.80; *Pf*  ^RA^ median: 0.93, IQR: 0.51–0.99). ***p < 0.0001 using Chi squared test.

Using tortuosity-based analysis we quantified pattern characteristics of sporozoite tracks. First, the individual experiments were evaluated (Sup. Fig. S1), thereafter the data of all *Pf*  ^RA^ and *Pf*  ^WT^ was pooled (Sup. Fig. S2) and further analyzed. Automated pattern classification of sporozoite tracks showed that 37% of *Pf*  ^WT^ sporozoite tracks were linear, 42% classified as sharp turn and 21% as slight turn (Fig. 3A). *Pf*^WT^ sporozoite tracks displayed a median SI of 0.86, indicating relatively straight tracks (i.e. indices close to 1; Fig. 3B) and a balanced AD of 0.45 indicating random meandering of sporozoites (AD close to 1 indicates a consistent track, AD close to 0 indicate random direction changes; Fig. 3C).

In contrast, the *Pf*  ^RA^ population displayed significantly more slight and sharp turn segments (7.2% blue and 74.3% yellow color coding respectively), and a decrease in linear patterns (18.5%, red) as compared to the *Pf*  ^WT^ (Fig. 3A). This difference was caused by continuous circular turning behavior of sporozoites (arrowheads Fig. 3A), as well as a back and forth motion (180° turn; hereafter termed “reversal”). Although *in vitro* on circular movement of *Pf* is reported on coated surfaces, under the conditions studied *Pf* did not show such movement (see Sup. Movie S4). This was confirmed by an increase in SI values close to 0, representing these turning tracks (Fig. 3B; post hoc Chi squared test p = 0.008 and p =  < 0.0001 respectively). In addition, *Pf*  ^RA^ sporozoites showed significantly more persistent straight tracks compared to *Pf*  ^WT^ i.e. increased indices close to 1 (Overall Chi squared test p =  < 0.0001; median SI 0.89). Similarly, *Pf*  ^RA^ showed more consistent tracks with fewer deviations from the mean angle of movement patterns compared to *Pf*  ^WT^ (Fig. 3C; angular dispersion median 0.92; overall Chi squared test p =  < 0.001 post hoc Chi squared test AD > 0.75 p =  < 0.0001). Taken together, the pattern analysis and tortuosity parameters indicate that *Pf*  ^RA^ exhibit reduced motility variation compared to *Pf*  ^WT^ and preferentially display continuous circling patterns.

### RA causes sporozoites to circle consistently in a clockwise direction

**Figure 4:**
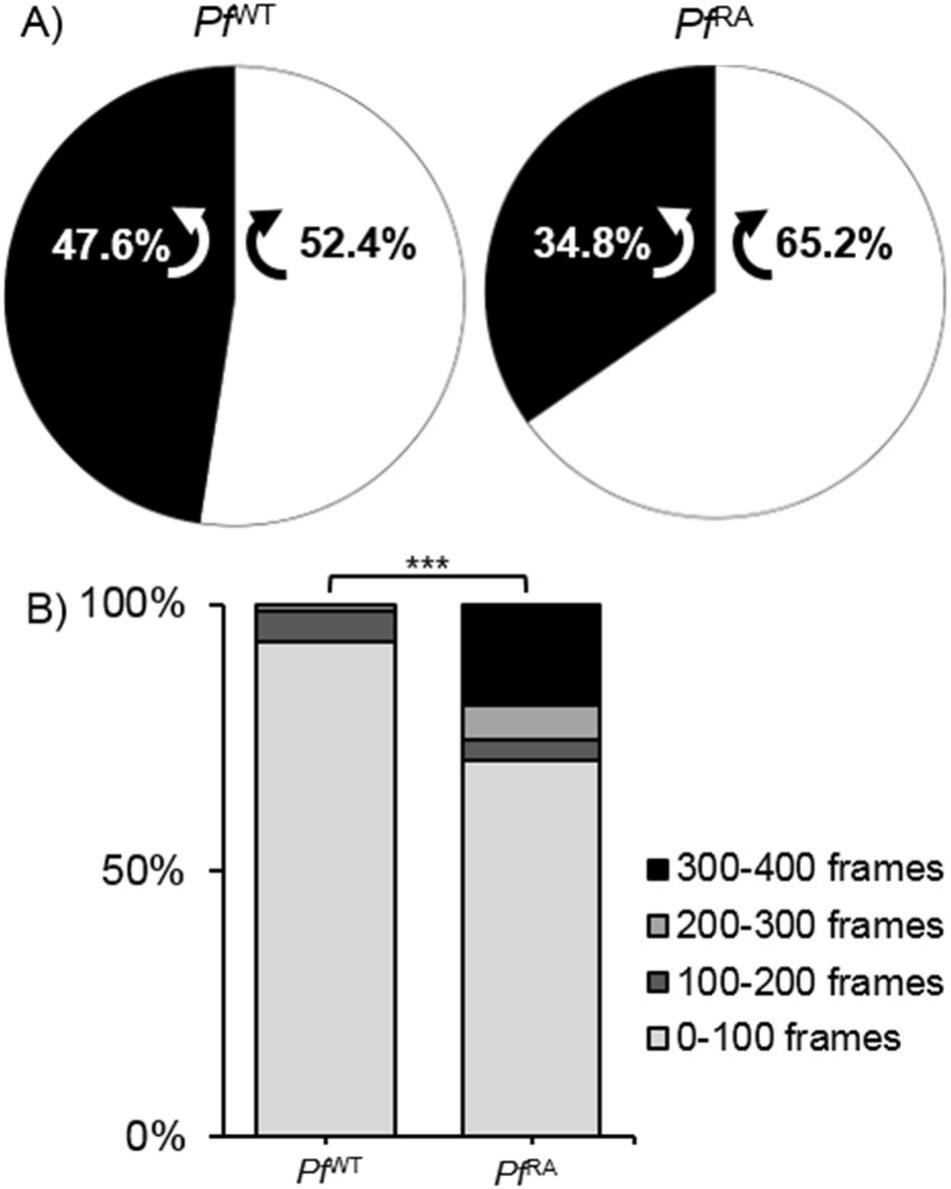
Direction of turning sporozoites. (**A**) Turn direction of *Pf*  ^WT^ and *Pf*  ^RA^ in skin. *Pf*  ^RA^ turn significantly more CW than *Pf*  ^WT^, p = 0.013 using Chi-Squared test. (**B**) The sharp turns of *Pf*  ^RA^ contain significantly more frames than the turns of *Pf*  ^WT^ (*Pf*  ^WT^ median: 32, IQR: 22–55; *Pf*  ^RA^ median: 33, IQR: 14–218; p > 0.000 using Chi-Squared test). Indicating persistent circular motion.

Possibly due to the tilted arrangement of the sporozoite polar-ring^33^, circularly moving sporozoites display a preferred clockwise (CW) turn direction *in vitro*^34^. In the three dimensional (3D) skin environment this preference was lost; SMOOT_human skin_ analysis demonstrated *Pf*  ^WT^ sporozoites turned equally CW and counterclockwise (CCW; Fig. 4A). Surprisingly, analysis of turn direction in the *Pf*  ^RA^ population yielded a preference for CW directionality (Fig. 4A; 65.2% CW; p = 0.013). Analysis of the duration of the turns (number of frames) revealed that *Pf*  ^RA^ sporozoites continued turning in circles when this pattern was initiated.

### Per frame analysis of velocity reveals decreased velocity alterations after RA

**Figure 5:**
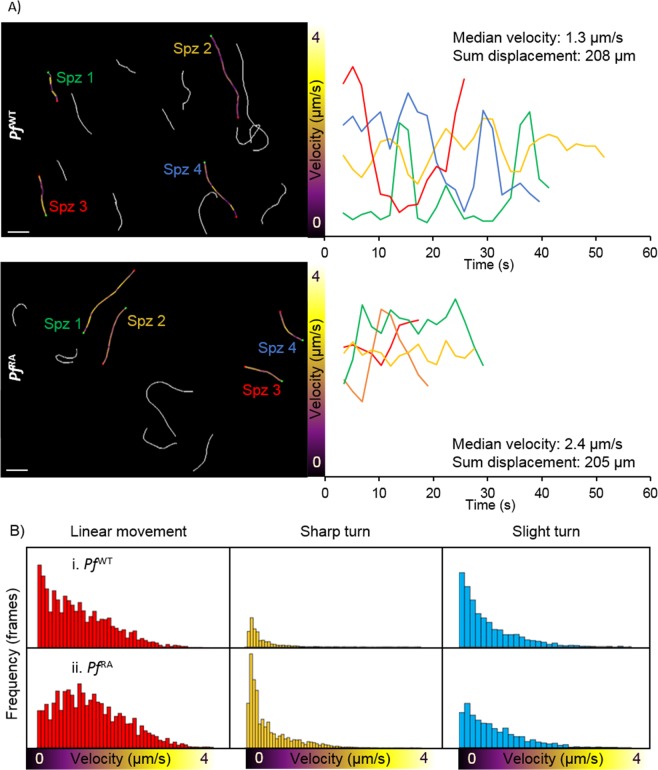
Velocity of sporozoites. (**A**) Four *Pf*  ^WT^ (above) and 4 *Pf*  ^RA^ (below) tracks are color-coded based on velocity. Scalebar: 20 μm. Their individual velocity is plotted over time (right). (**B**) Velocity distribution of *Pf*  ^WT^ and *Pf*  ^RA^ per movement pattern.

In line with previous findings^9,10,35^, we recorded an average sporozoite velocity per track of 1.1 µm/s (time did not seem to have an effect on the average velocity (Sup. Fig. S3)). SMOOT_human skin_ also allowed analysis of the velocity per captured frame within a track (Fig. 5), revealing marked variations over time. Examples illustrated in Fig. 5A show a single sporozoite can display non-parametric velocity changes between 0 and 3.5 µm/s over the course of one track (Fig. 5A upper panel; Spz 3, red). While the velocity changes occurred within all movement patterns, the median velocity was highest in linear segments followed by slight turns and in sharp turns (Fig. 5B). *Pf*  ^RA^ consistently showed higher velocity in all movement patterns (median 1.1 µm/s vs 0.85 µm/s for linear tracks, 0.6 µm/s vs 0.48 for slight turns and 0.34 µm/s vs 0.24 for sharp turns in *Pf*  ^RA^ vs *Pf*  ^WT^ respectively, p =< 0.0001). Despite the fact that *Pf*  ^RA^ displayed more “slow” sharp turns, in a per frame analysis its overall median velocity at 0.37 µm/s was higher than the median velocity of *Pf*  ^WT^ (0.35 µm/s; p =< 0.0001). This difference was caused by a reduction in stop-and-go action (frames with velocity < 0.5 µm/s were 56.8% for *Pf*  ^RA^ and 59.6% for *Pf*  ^WT^, p = 0.037). Furthermore, velocity variability was smaller in *Pf*  ^RA^ (Fig. 5A, range 0–4.1 µm/s) compared to *Pf*  ^WT^ (0–4.8 µm/s). Corroborating this finding, *Pf*  ^RA^ velocities of linear tracks were normally distributed compared to a nonparametric velocity distribution for *Pf*  ^WT^ (Fig. 5C, p =  < 0.0001), whereas velocities in other movement patterns were nonparametrically distributed for both *Pf*  ^RA^ and *Pf*  ^WT^. Taken together, *Pf*  ^RA^ less readily alternated their velocity.

### ***Pf***  ^RA^ display a default motility pattern: reversal

**Figure 6:**
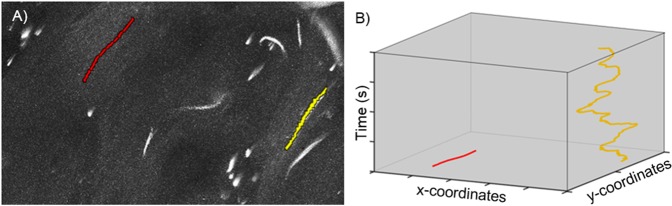
Reverse movement. (**A**) In this example, one sporozoite was moving in a linear direction and one sporozoite was moving back and forth. The linear track is depicted in red, the reverse movement is classified as a sharp turn, thereby depicted in yellow. (**B**) The coordinates of the linear and the reversal track are plotted in a xyt coordinate axis system.

Interestingly, some sporozoites in the *Pf*  ^RA^ group (9% of motile *Pf*  ^RA^ tracks) revealed pendulum movement, whereby they reverse direction repeatedly moving up and down a short path (Fig. 6; Sup. Movie S3). This is in line with earlier findings where sporozoites moved in this particular fashion while residing in the mosquito^36–38^. Plotting this movement (over x and y axis) over time yielded a sinuous track (yellow) that is clearly distinct from the short linear track (red) observed in the same movie segment. Strikingly, this movement pattern was not observed in *Pf*  ^WT^.

## Discussion

In this study we compared movement of wild-type with irradiated *Pf* malaria sporozoites in a human skin explant using our semi-automated custom analysis tool for sporozoite tracking. We found that *Pf*  ^RA^ display increased circular motility patterns, more extreme SI values and higher AD compared to *Pf*  ^WT^. In addition, *Pf*  ^RA^ exhibit less variability in velocity over the course of their track and “reversal” patterns were unique to this group. Combined, the data indicates that attenuation via radiation may alter sporozoite motility.

*In vitro*, sporozoites display very elementary circular movement with little variation in velocity, angle or direction^34,39,40^. Recently, we reported that for *Pb* sporozoites a complex interplay of various nutrients including albumin, glucose and certain amino acids and vitamins regulates parasite motility *in vitro*^41^. Under the conditions used to study the movement in human skin explants (uncoated glass; RPMI 160 + 10% FCS) *Pf* sporozoites did not display this movement pattern *in vitro* (Sup. Movie S4). In skin, however, sharp turns (combination of circular and reversal movement) were observed in addition to linear and slight turn movement patterns. This indicates that the environment influences the *Pf* sporozoite movement and in particular its directionality. It was previously shown for *Pb* sporozoites that an environment with artificial physical constraints in the form of pillars increases the movement directionality as function of interstitial space^27^. This trend is in line with the reported increase of tortuosity in porous materials that occurs when reducing the interstitial space^42^. An influence of tissue structure on directionality of *Pb* sporozoites can also be extrapolated from the different motility patterns in the skin of mouse ears as compared to the skin of a mouse tail^10^. Our findings suggest that the highly complex heterogeneity of human skin composition, in combination with the mixed nutrient availability, may similarly impact motility patterns of individual sporozoites, generating highly complex movement patterns which vary according to the local donor tissue structure.

Although *Pf* sporozoites display average track velocities in line with previous reports (average 1.1 µm/s as compared to previous studies reporting averages of 0.9–1.5 µm/s in mouse skin^9,10,35^), a per frame velocity analysis revealed ongoing stop-and-go actions (see Fig. 5). These heterogeneous patterns in combination with the high level of path tortuosity suggests that environmental ques and cellular interactions such as traversal^43^ impact heterogeneity of direction and speed of the sporozoites. Indeed, the sporozoite surface displays many proteins such as CSP, SPECT1 and CelTOS which facilitate interaction with targets like heparan sulphate and αvβ3 integrin which are available in the human skin^11,44,45^. Because these alterations in velocity result in a nonparametric velocity distribution which is different for sporozoites taking sharp or slight turns or moving straight, we reason that average velocity alone is insufficient to unravel complex migratory behavior.

Our findings suggest that radiation may not only effectively attenuate *Pf* sporozoites at the liver stage, which has been described before^8^, but could also influence their motility at the skin stage. Similar to what has been described before with respect to the effect of cryopreservation on *Pb* sporozoite motility^46^, RA also seems to induce small alterations in motility. The differences in motility between *Pf*  ^WT^ and *Pf*  ^RA^ (observed in both donors), although minor, are indicative of a reduced complexity of the *Pf*^  *RA*^ interaction with the tissue environment. Moreover, the increased AD of *Pf*  ^RA^, and the increased duration of their turns seems to suggest that once a “default” movement pattern has been initiated, the movement pattern persists. As circling and reversal patterns mean the *Pf* sporozoites stay in a single location, one could argue this movement pattern would render parasites at risk for elimination by phagocytic dermal immune cells, which could impact antigen presentation and vaccine efficacy.

Understanding how radiation attenuation interferes with these pathogen-host interactions could be important to identify novel vaccine targets or improve the efficacy of existing radiation attenuated sporozoite vaccines. Here it should be noted that for our studies we solely used 20Krad attenuation dose as described previously in murine studies^47,48^, meaning we cannot state if a similar difference would be observed at lower radiation doses. However, increased radiation dosages result in reduced infectivity of *Plasmodium* species and reduced effectivity of attenuated parasite immunization^8,49^. The altered motility induced by radiation may contribute to this reduced infectivity. Whether similar effects also occur when using genetically modified sporozoites vaccines^50–52^, remains to be investigated.

Use of viable human skin explants allowed us to analyze *Pf* sporozoite movement in their natural skin environment thereby enhancing the possibilities to gain insight in their behavior. Obviously, also this model system has limitations. Although not per se relevant for the field of live attenuated *Pf* vaccines, intradermal syringe-based injections may not accurately represent the mosquito based transmission of the disease^53^. The lack of blood and lymphatic circulation limited prevents *Pf* to migrate out of the skin, which means the motility of the total population of administered *Pf* is analyzed. Due to the light attenuation of tissue the analysis of sporozoite movement was restricted to 2D, which shortened the length of the more linear tracks and thus biased circular and reversal movement patterns occurring in plane. Finally, the location in the dermis that was imaged seemed to effect the sporozoite movement patterns (Sup. Fig. S1) even when the same batch of *Pf* was used. Nevertheless, we feel confident that the analysis performed in *ex vivo* human skin helps building a bridge between *in vitro* assays and *in vivo* assays of *Pb* sporozoites in mouse skin^9,10^ and controlled human infection studies^21,50^.

In conclusion, we imaged *Pf* sporozoite migration in the dermis of its natural host and performed an in-depth analysis of the motility of WT and RA *Pf* sporozoites. We demonstrate loss of movement variability after radiation attenuation which might reflect reduced viability and ultimately decreased infectivity. Because of the ability of SMOOT_human skin_ to analyze complex migration, it may contribute to the refinement of live sporozoite vaccine formulations.

## Materials and Methods

### Study design

In order to explore movement characteristics of *Pf* sporozoites in human skin explants, we performed a controlled laboratory experiment, in which we compared motility parameters (outputted by our custom software SMOOT_human skin_, see below) of unattenuated *Pf* parasites *Pf* ^WT^: 352 sporozoites (14 movie sections of 400 frames/11 min) with those of radiation attenuated *Pf* parasites *Pf* ^RA^: 350 sporozoites (18 movie sections 400 frames/11 min). The movies were made of sporozoites injected in skin explants of two donors (in two independent experiments), comparing *Pf* ^WT^ with *Pf* ^RA^ in both donors at two different locations in the skin ending up with four unique locations for both *Pf* ^WT^ and *Pf* ^RA^. The experiments were evaluated individually (Sup. Fig. S1), thereafter the data was pooled (Sup. Fig. S2) and the final analysis of the motility of *Pf* ^WT^ vs *Pf* ^RA^ was performed on the pooled dataset. The study was not randomized and not blinded.

### Parasites

*Anopheles stephensi* mosquitoes infected with a transgenic *Pf* line that constitutively expresses fluorescent reporter protein GFP under the *pf*CS promotor (M.W. Vos *et al*., manuscript in preparation), were killed using ethanol spray and rinsed in RPMI 1640 (Invitrogen, Carlsbad, CA, USA). Salivary glands were dissected manually at day 14–21 post infection, incubated in RPMI 1640 and kept on ice. Radiation attenuation of sporozoites was performed by irradiating intact salivary glands to a total dose of 20 krad using a Cesium radiation source (total of 28 minutes) on ice. During this time control sporozoites were also kept on ice. Within one-hour, glands were homogenized to release *Pf* sporozoites. Sporozoites were then counted using a Burker chamber, brought to a concentration of 20 × 10^6^/ml in RPMI 1640 containing 10% Fetal Calf Serum (FCS; Bodinco, Alkmaar, The Netherlands) and used for imaging experiments immediately.

### Skin explants

We obtained human skin explants from collaborating hospitals immediately after abdominal skin reduction surgery (CME B18.009) and kept skin explants at 4 °C for 3 hours until use. Subcutaneous fat was removed and the epidermal side was cleaned with 70% ethanol. One million sporozoites were injected intradermally in a 50 μl injection using a 0.3 ml insulin syringe (30 G; BD, Franklin Lakes, NJ, USA). In order to facilitate quick localization of the injection site by confocal microscopy the injection formulation contained Yellow-Green fluorescent 500 nm Latex nanoparticles (Sigma Aldrich). Immediately after injection, the injection site was biopsied using a 6 mm biopsy punch, sliced longitudinally through the center and mounted on a microscopy slide with a 1 mm depression in RPMI 10% FCS. Slides were imaged within 30 minutes post injection.

### Confocal video-microscopy

Skin biopsy slides were imaged using the time-lapse function of the Leica TSC SP8 Confocal microscope (Leica, Wetzlar, Germany) at a temperature of 37 degrees Celsius, 5% CO_2_. 2D images (no z-stacks) were obtained using an exposure time of 1.7 seconds per frame and a 40x objective (400 frames per movie, 11 minutes). Microscopy videos were rendered using accompanying Leica LASX software and were analyzed using custom software SMOOT_human skin_.

### SMOOT_human skin_

MATLAB (The MathWorks Inc. Natick, MA, USA) software was created for in skin sporozoite analysis, which we called Sporozoite Motility Orienting and Organizing Tool (SMOOT_human skin_). This tool is an extended version compared to the SMOOT_human skin_ tool previously used to determine the velocity and movement pattern distribution of Cy5M_2_ labeled *Pf* sporozoites^54^. Similar to our recently published *in vitro* tool SMOOT_*In vitro*_^41^, the upgraded SMOOT_human skin_ software now also includes turn angle and displacement. In addition, SMOOT_human skin_ takes into account the directionality by computing: angular dispersion, straightness index and the direction of sporozoite tracks. Firstly, sporozoite tracks were characterized as motile or stationary based on their displacement. Subsequently, motile tracks were subdivided into movement patterns: *sharp turn, slight turn* and *linear*.

To investigate the influence of RA on sporozoite motility, we compared SMOOT_human skin_ parameter outcomes of 14 *Pf*  ^WT^ motility movie files (11 minutes/movie, 154 minutes total, 352 sporozoite tracks consisting of 511 segments and 26932 frames; Sup. Movie S1) with 18 *Pf*  ^RA^ motility files (11 minutes/ movie, 198 minutes total, 350 sporozoite tracks consisting of 563 segments and 25804 frames; Sup. Movie S2). Software output was manually validated.

Velocity was determined by measuring the displacement between frames. We defined step number in the track *i* to measure velocity *v* using formula (1), with *x as* the median pixel location of the segmented structure and *t* as the time passed in seconds.

1 $${\boldsymbol{v}}({\boldsymbol{i}})=\frac{{{\boldsymbol{x}}}_{{\boldsymbol{i}}}-{{\boldsymbol{x}}}_{{\boldsymbol{i}}-1}}{{{\boldsymbol{t}}}_{{\boldsymbol{i}}}-{{\boldsymbol{t}}}_{{\boldsymbol{i}}-1}}=\frac{{\boldsymbol{dx}}}{{\boldsymbol{dt}}}$$

The mean squared displacement (MSD) is a common measure to distinguish random versus non-random motion for moving particles and was previously used to analyze sporozoite motility^5,6^. The squared displacement (SD) is a measure of the displacement per time point of an individual track, which can be calculated with formula (2):

2 $${\boldsymbol{SD}}({\boldsymbol{i}})={({{\boldsymbol{x}}}_{{\boldsymbol{n}}}({\boldsymbol{i}})-{{\boldsymbol{x}}}_{{\boldsymbol{n}}}(0))}^{2}$$

The MSD was derived from the SD of all linear tracks using formula (3):

3 $${\boldsymbol{MSD}}({\boldsymbol{i}})={({\boldsymbol{x}}({\boldsymbol{i}})-{{\boldsymbol{x}}}_{0})}^{2}=\frac{1}{{\boldsymbol{N}}}\mathop{\sum }\limits_{{\boldsymbol{n}}=1}^{{\boldsymbol{N}}}{({{\boldsymbol{x}}}_{{\boldsymbol{n}}}({\boldsymbol{i}})-{{\boldsymbol{x}}}_{{\boldsymbol{n}}}(0))}^{2}$$

The turn angle (θ) of the sporozoite was defined by the angle difference between path directions in consecutive frames. If we start calculating the turn angle from location *x*_0_ then the sporozoite reaches *x*_*i*_ after *i* steps. The angle of *x*_*i*_ is the angle *δ*_*i*_ between point x_*i*_ and the horizontal. The turn angle was then defined as described in formula (4).

4 $${{\boldsymbol{\theta }}}_{{\boldsymbol{i}}}={{\boldsymbol{\delta }}}_{{\boldsymbol{i}}}-{{\boldsymbol{\delta }}}_{{\boldsymbol{i}}-1}$$

The straightness index (SI) is the most basic approach to quantify tortuosity and is defined as the ratio of distance between track end points (*C*) and track length (*L*), as calculated using formula (5). This parameter quantifies deviation from a straight line, e.g. SI = 1 in a perfect linear path, SI = 0 in circular motion.

5 $${\boldsymbol{St}}=\frac{{{\boldsymbol{C}}}_{{\boldsymbol{track}}}}{{{\boldsymbol{L}}}_{{\boldsymbol{track}}}}=\frac{{\boldsymbol{x}}({\boldsymbol{i}})-{\boldsymbol{x}}(0)}{{\sum }_{{\boldsymbol{k}}=1}^{{\boldsymbol{i}}}({\boldsymbol{x}}({\boldsymbol{k}})-{\boldsymbol{x}}({\boldsymbol{k}}-1))}$$

Angular dispersion (AD) quantifies the number of turning angles diverging from the main angle of movement. It describes tortuosity by quantifying changes in direction. It is calculated using the turn angles (formula (4)) according to the following formula:

6 $${\boldsymbol{AD}}=\frac{1}{{\boldsymbol{I}}}\sqrt{{{\boldsymbol{C}}}^{2}+{{\boldsymbol{S}}}^{2}}$$

Where *I* is the last step of the track and *C and S* are defined as:

7 $${\boldsymbol{C}}=\mathop{\sum }\limits_{{\boldsymbol{i}}=1}^{{\boldsymbol{I}}}\,\cos \,{{\boldsymbol{\theta }}}_{{\boldsymbol{i}}}\,{\boldsymbol{S}}=\mathop{\sum }\limits_{{\boldsymbol{i}}=1}^{{\boldsymbol{I}}}\,\sin \,{{\boldsymbol{\theta }}}_{{\boldsymbol{i}}}$$

### Statistics

Data extracted from SMOOT_human skin_ was analyzed in IBM (Armonk, NY, USA) SPSS version 23 or GraphPad Prism (La Jolla, CA, USA) version 7. Comparisons between two or more independent categorical data groups were made by Chi-squared test, continuous nonparametric parameters were compared by Mann-Whitney U test. P < 0.05 was considered statistically significant. Bonferroni correction was applied for post hoc analysis after Chi-squared testing.

### Ethics statement

The use of human skin explants (obtained as waste material after abdominal reduction surgery) for this research was approved by the Commission Medical Ethics (CME) of the LUMC, Leiden. Approval number CME: B18–009. The methods were carried out in accordance with the relevant guidelines and regulations. Informed consent was obtained from all participants.

## Supplementary information

Movie S1. Example of a confocal microscopy movie showing PfWT sporozoites migrating through human skin explant tissue.

Movie S2. Example of a confocal microscopy movie showing PfRA sporozoites migrating through human skin explant tissue.

Movie S3. Example of reversal movement exhibited by PfRA sporozoites.

Movie S4. Example of PfWT sporozoite motility on uncoated glass surfaces.

Supplementary Information

## Data Availability

The data can be made available upon request
