# Supplementary material for: Quantification of wild-type and radiation attenuated Plasmodium falciparum sporozoite motility in human skin
Source: Sci Rep. 2019 Sep 17;9:13436. doi: 10.1038/s41598-019-49895-3 (PMC6748968; doi:10.1038/s41598-019-49895-3)
Supplement: Supplementary file 5 — Supplementary Information [file 41598_2019_49895_MOESM5_ESM.pdf]

Quantification of wild-type and radiation attenuated *Plasmodium falciparum* sporozoite motility in human skin

Béatrice M.F. Winkel[1,2]*, Clarize M. de Korne[1,2]*, Matthias N. van Oosterom[2], Diego Staphorst[2], Mark Meijhuis[2], Els Baalbergen[1], Munisha S. Ganesh[1], Koen J. Dechering[3], Martijn W. Vos[3] Séverine C. Chevalley-Maurel[1], Blandine Franke-Fayard[1], Fijs W.B. van Leeuwen[2], Meta Roestenberg[1,4§]

*These authors contributed equally tot his work

[1]Department of Parasitology, Leiden University Medical Center, Albinusdreef 2, 2333 ZA Leiden, The Netherlands

[2]Interventional Molecular Imaging laboratory, Department of Radiology, Leiden University Medical Center, Albinusdreef 2, 2333 ZA Leiden, The Netherlands

[3]TropIQ Health Sciences, Transistorweg 5, 6534, Nijmegen, The Netherlands

[4]Department of Infectious Diseases, Leiden University Medical Center, Albinusdreef 2, 2333 ZA Leiden, The Netherlands

§To whom correspondence should be addressed: M. Roestenberg, Leiden University Medical Center (LUMC), department of Parasitology, Albinusdreef 2, 2333 ZA Leiden, The Netherlands; Tel: + 31 71 5264400; Fax: + 31 71 5266907; email: m.roestenberg@lumc.nl.

SUPPLEMENTARY INFORMATION

Figure S1. Overview sporozoite tracks for individual locations.

Figure S2. Pooling process of sporozoite motility data.

Figure S3. Sporozoite motility over time.

Movie S1. Example of a confocal microscopy movie showing *Pf*^{WT} sporozoites migrating through human skin explant tissue.

Movie S2. Example of a confocal microscopy movie showing *Pf*^{RA} sporozoites migrating through human skin explant tissue.

Movie S3. Example of reversal movement exhibited by *Pf*^{RA} sporozoites.

Movie S4. Example of *Pf*^{WT} sporozoite motility on uncoated glass surfaces.

Evaluation and pooling of individual experiments

Two independent experiments were performed to investigate the effect of radiation attenuation on sporozoite motility. During each experiment sporozoites were obtained by dissection and half of them were radiated to obtain a Pf^{WT} and Pf^{RA} sample. One million Pf^{WT} and Pf^{RA} were injected intradermally in a skin explant from the same donor and imaged by confocal microscopy, both samples at two different locations. The movies made at the 4 different locations per donor were divided in sections of 400 frames, because the length of the movies varied from 800-2000 frames and SMOOT$_{\text{human skin}}$ is optimized to process movies of 400 frames.

The color-coded track overviews of the movies made at the eight different locations are shown in supplementary figure 1. Linear segments are depicted in red, slight turns in blue and sharp turns in yellow. The latter consisted of both sporozoites moving forward while turning sharply and sporozoites moving back and forth (pendulum movement) making a 180° turn (see also Fig. 6 for more detailed explanation of this movement pattern). In the overviews these tracks seem linear but are coded “yellow”, because of their 180° sharp turn. The movement pattern distribution of the Pf^{WT} and Pf^{RA} was quantified at frame level. In both donors more sharp turns were found in the Pf^{RA} sample. In donor 1 Pf^{WT} did make some sharp turns and one sporozoite started circling, while Pf^{RA} made more sharp

turns in the form of reversal movement (Sup. Fig. 1a-d). In donor 2 the Pf^{RA} exhibited the circling movement, which is observed *in vitro* (Sup. Fig. 1g-h). The difference in movement pattern distribution seen for the Pf^{RA} in donor 2 (Sup. Fig. 1g-h) revealed the effect of location on movement pattern and underlined that the location needs to be standardized as much as possible by using the same part of the skin and imaging at the same depth.

The data from the eight different locations was pooled before the detailed motility analysis was performed which is reported in the main manuscript. Supplementary figure 2 shows the effect of pooling per donor and the effect of pooling the data from both donors. The same trend is visible when comparing the movement pattern distribution of Pf^{WT} and Pf^{RA} per donor or after pooling the data from both donors.

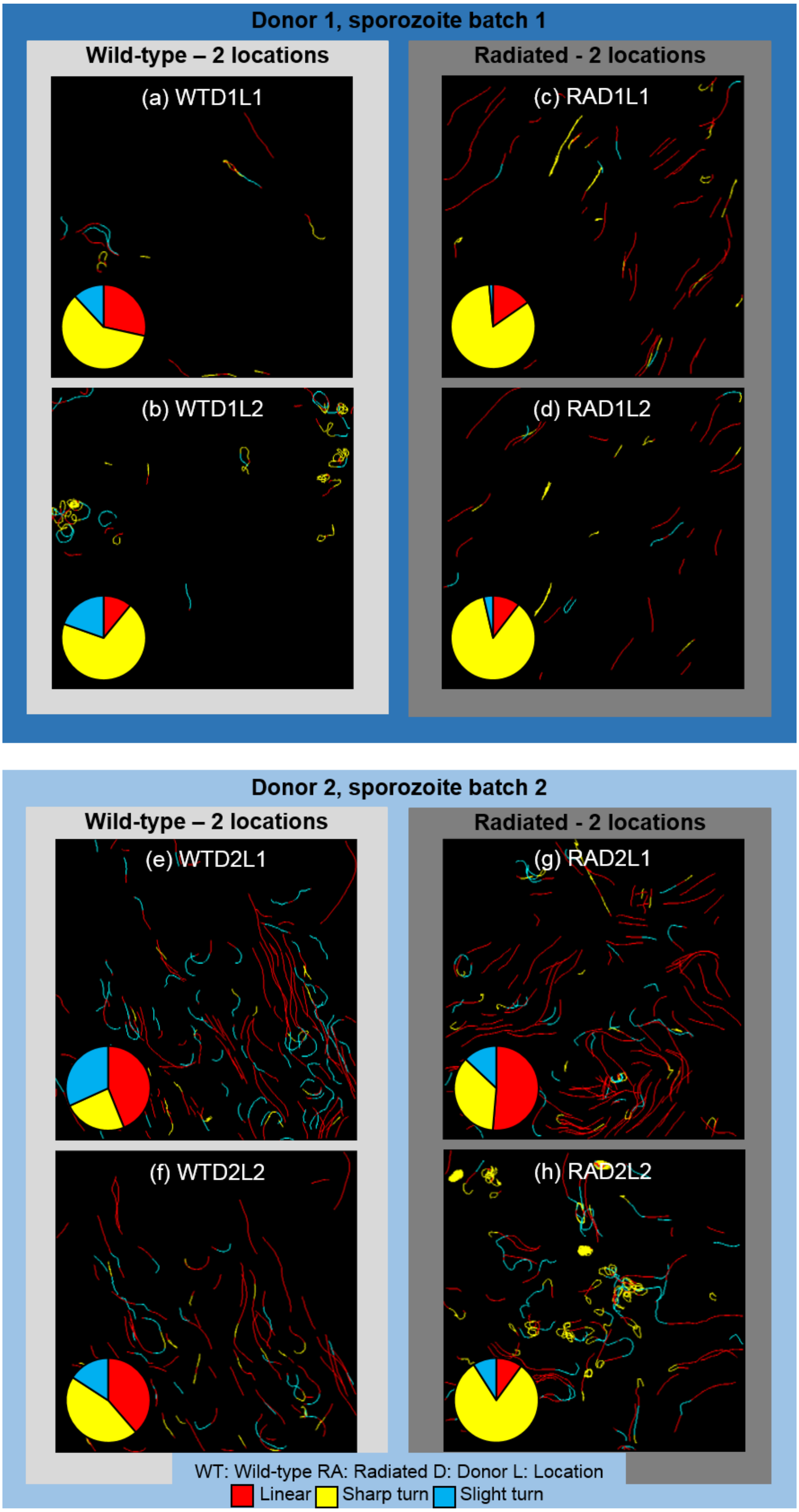

Donor 1, sporozoite batch 1
Wild-type – 2 locations
(a) WTD1L1
(b) WTD1L2
Radiated - 2 locations
(c) RAD1L1
(d) RAD1L2
Donor 2, sporozoite batch 2
Wild-type – 2 locations
(e) WTD2L1
(f) WTD2L2
Radiated - 2 locations
(g) RAD2L1
(h) RAD2L2
WT: Wild-type RA: Radiated D: Donor L: Location
Linear
Sharp turn
Slight turn

Sup. Fig. 1 Overview sporozoite tracks for individual locations

(a-b) Track overview and movement pattern distribution for wild-type sporozoites injected into the skin explant from donor 1 and imaged at two different location. **(c-d)** Track overview and movement pattern distribution for radiation attenuated sporozoites injected into the skin explant from donor 1 and imaged at two different location. **(e-f)** Track overview and movement pattern distribution for wild-type sporozoites injected into the skin explant from donor 2 and imaged at two different location. **(g-h)** Track overview and movement pattern distribution for radiation attenuated sporozoites injected into the skin explant from donor 2 and imaged at two different location. The field of view was 290x290 µm for all movies.

Pooling of individual experiments

The data from the eight different locations was pooled before the detailed motility analysis was performed which is reported in the main manuscript. Supplementary figure 2 shows the effect of pooling per donor and the effect of pooling the Pf^{WT} and Pf^{RA} data from both donors.

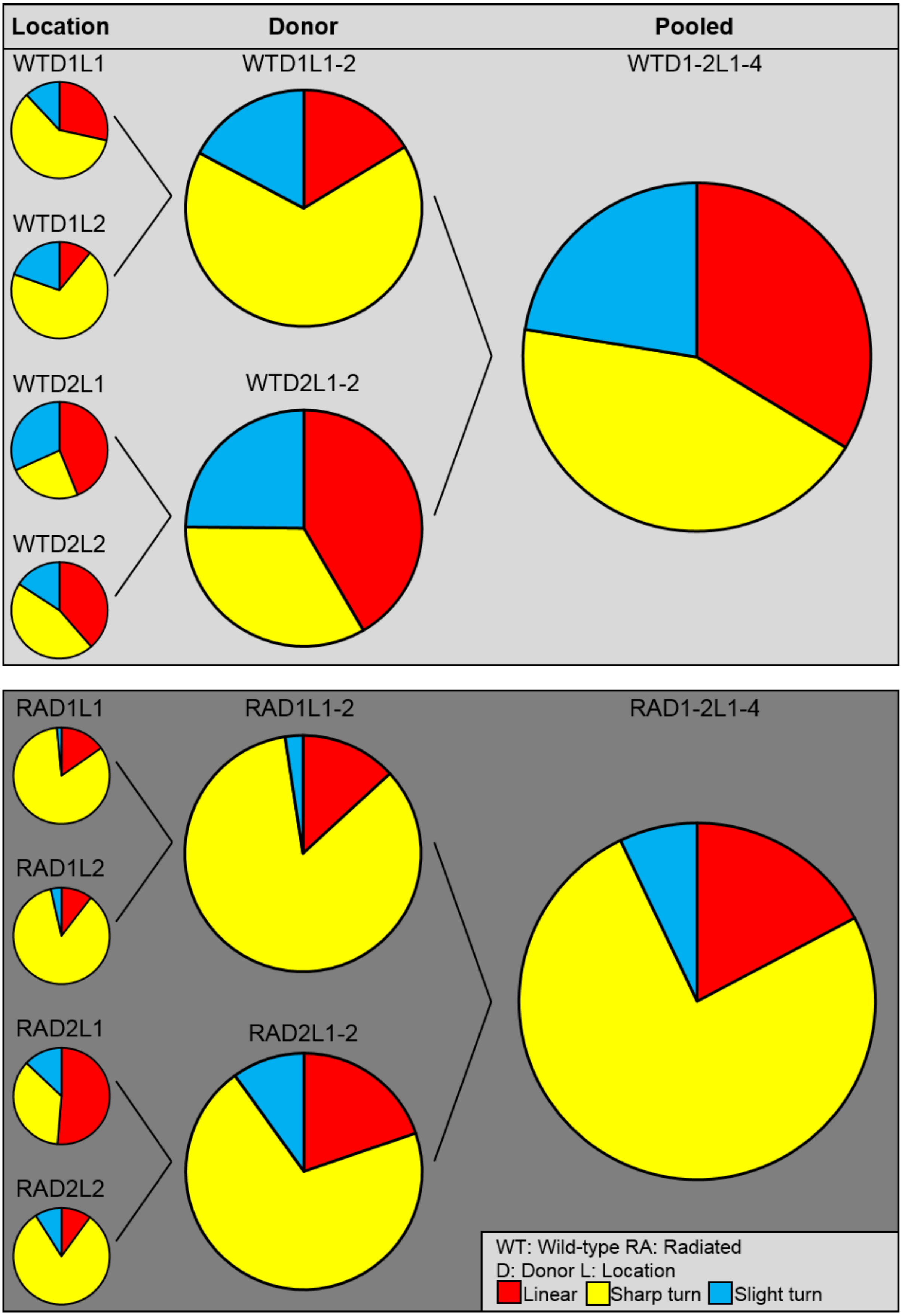

Location
Donor
Pooled
WTD1L1
WTD1L1-2
WTD1-2L1-4
WTD1L2
WTD2L1
WTD2L1-2
WTD2L2
RAD1L1
RAD1L1-2
RAD1-2L1-4
RAD1L2
RAD2L1
RAD2L1-2
RAD2L2
WT: Wild-type RA: Radiated
D: Donor L: Location
Linear
Sharp turn
Slight turn

Sup. Fig. 2 Pooling process of sporozoite motility data

The movement pattern distribution is shown for wild-type (WT) and radiation attenuated (RA) sporozoites per individual location (L), per donor (D) and after pooling. The movement pattern distribution of radiation attenuated sporozoites was significantly different from the movement pattern distribution of wild-type sporozoites, both per donor and after pooling ($p<0.0001$; Chi-Squared test).

In vitro assay with Plasmodium falciparum

For the *in vitro* assay, the sporozoites were obtained in the same way as for the experiments in human skin explants. For imaging of the sporozoites, 10 µl of the spz solution was pipetted on the cover slip of a confocal dish without any precoating (ø14mm; MatTek Corporation), covered with another cover slip (ø12mm; VWR Avantor) and imaged within half an hour (Sup. Movie 4).

Sporozoite velocity and movement pattern distribution over time

To assess the influence of the time on sporozoite velocity we longitudinally sampled 1 movie file over a 2h time period, see Sup. Figure 3. This data suggested that in the current setting time had little influence on the velocity of *Plasmodium falciparum* (trendline slope: decrease of 0.0023 µm/sec per minute; R^2: 0.3). In the same movie we assessed the influence of time on the turning behavior of sporozoites and again, there seem to be little to no influence (trendline slope: decrease of 0.09 percent per min; R^2: 0.4).

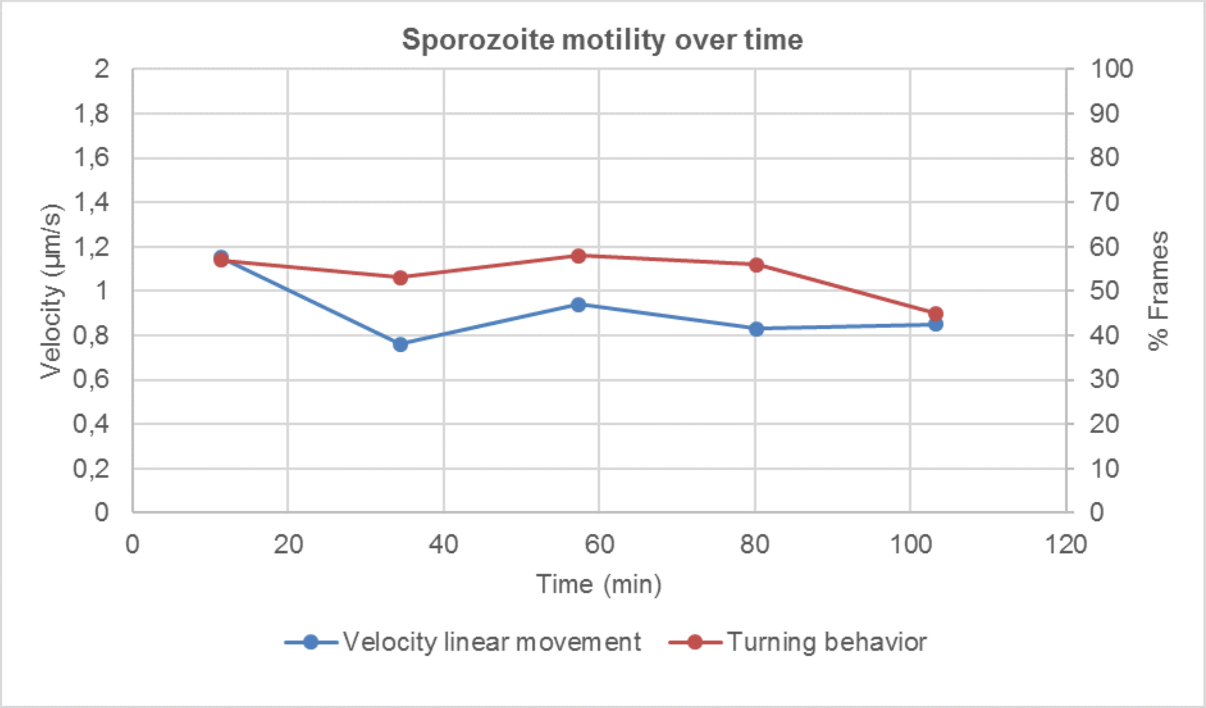

Sup. Fig. S3 Sporozoite motility over time

A 4000 frames movie file was longitudinally sampled in 5 sections of 800 frames (23 min). The velocity of the sporozoites during linear movement is depicted in blue and the percentage of turns (calculated at frame level) is depicted in red.
